# Comparative study of enteric viruses, coliphages and indicator bacteria for evaluating water quality in a tropical high-altitude system

**DOI:** 10.1186/1476-069X-8-49

**Published:** 2009-10-27

**Authors:** Ana C Espinosa, Carlos F Arias, Salvador Sánchez-Colón, Marisa Mazari-Hiriart

**Affiliations:** 1 Instituto de Ecología, Universidad Nacional Autónoma de México, Circuito Exterior, Ciudad Universitaria, Coyoacán, 04510 México, DF, México; 2 Instituto de Biotecnología, Universidad Nacional Autónoma de México, Av Universidad 2001, Col Chamilpa, 62210 Cuernavaca, Morelos, México; 3 Consultoría Ambiental y Estadística, Cerrada de Cortés 43, Colonia Campestre Tlacopac, San Ángel, 01049 México, DF, México

## Abstract

**Background:**

Bacteria used as indicators for pathogenic microorganisms in water are not considered adequate as enteric virus indicators. Surface water from a tropical high-altitude system located in Mexico City that receives rainwater, treated and non-treated wastewater used for irrigation, and groundwater used for drinking, was studied.

**Methods:**

The presence of enterovirus, rotavirus, astrovirus, coliphage, coliform bacteria, and enterococci was determined during annual cycles in 2001 and 2002. Enteric viruses in concentrated water samples were detected by reverse transcriptase-polymerase chain reaction (RT-PCR). Coliphages were detected using the double agar layer method. Bacteria analyses of the water samples were carried out by membrane filtration.

**Results:**

The presence of viruses and bacteria in the water used for irrigation showed no relationship between current bacterial indicator detection and viral presence. Coliphages showed strong association with indicator bacteria and enterovirus, but weak association with other enteric viruses. Enterovirus and rotavirus showed significant seasonal differences in water used for irrigation, although this was not clear for astrovirus.

**Conclusion:**

Coliphages proved to be adequate faecal pollution indicators for the irrigation water studied. Viral presence in this tropical high-altitude system showed a similar trend to data previously reported for temperate zones.

## Background

At present, public health concerns remain focused on waterborne diseases, with incidence data in both developed and developing countries making gastroenteritis highly important. A diversity of enteric bacteria and viruses has been associated with outbreaks of waterborne gastroenteritis [1,2].

Since the late 19th century, bacteria have been used as indicators of water quality [3]. Although there are reports concerning the inadequacy of bacteria as microbiological water quality indicators [4], it has been recognized that they are indicators of a broad bacterial group and regular human microbiota [5]. Nevertheless, bacteria alone offer limited information regarding microbiological water quality as they do not reflect the presence of enteric viruses or protozoa [6].

The presence of viruses and other pathogens in the environment is an indicator of faecal pollution that poses a potential risk to the exposed population, since such pathogens do not constitute normal gastrointestinal microbiota, and are only excreted by sick individuals [7]. Rotavirus is recognized as being responsible for diarrheal disease in young children with a worldwide mortality rate of 600,000 per year [8]. Astrovirus is also considered one of the most important agents of viral gastroenteritis [9,10], and is ranked second after rotavirus as the major cause of diarrheal disease in young children and adults [11]. While the actual contribution of rotavirus to total incidence of diarrheal disease is between 25% and 52%, astrovirus is much lower being responsible for between 5% and 10% of cases [12].

In Mexico, during autumn and winter, which correspond to the cold-dry season, rotavirus has been reported as the main etiologic agent of diarrheal disease in children aged two years and under, and as has already been mentioned, it is responsible for approximately 25%-50% of all gastrointestinal cases [13,14]. Epidemiological studies show a seasonal incidence of bacterial diarrheal disease mainly during the summer months that coincides with the warm-rainy season [15-17].

It is important to consider enteric viruses in water quality studies not only because of their incidence as causal agents for diarrheal disease [8,13], but also due to their characteristics, which allow them to survive in the environment for long periods of time, and tolerate changing environmental conditions [4,18].

Although it is not possible to establish a direct relationship between epidemiological and environmental data, it is important to consider microbial water quality in terms of water use. Furthermore, it is important to assess the potential risk to the exposed population, especially in developing countries, considering that recycled water has been associated with the presence and re-emergence of waterborne diseases worldwide [19].

Mexico is one of the main countries that reuse wastewater for irrigation of land used for crop cultivation, an area which has been calculated to be approximately 180,000 ha [20]. This practice is likely to increase, and therefore, it would be advisable to assess water quality in terms of both viral composition and load in order to decrease the associated risk to the population. For such assessment it would be necessary to evaluate the most adequate indicator from a public health perspective, whether it is bacterial or viral.

The aim of this study was to compare the presence of enterovirus, rotavirus, astrovirus, coliphages and indicator bacteria in a tropical high-altitude system, which supplies the Southern area of Mexico City with water for both irrigation and drinking.

## Methods

The study area is located in the South of Mexico City. It is a tropical high-altitude aquatic system located at 2240 masl, between 19°02 and 20°12' N and 98°28' and 99°32' W, covering an area of 1,479 km^2^. The average annual temperature is 16°C, but the temperature fluctuates greatly during the day with an average maximum of 25°C and minimum of 8°C. The rainy season mainly occurs during the summer and autumn months (May to October), while the rest of the year remains dry.

Agriculture and farming remain the main activities in this area with water being pumped from the canal network for surface irrigation. Flowers and vegetables are cultivated in the area with some of the latter being eaten raw. There are some domestic animals, as well as "conservation areas" that have been invaded by squatter settlements, a common practice as part of the urbanization process in developing countries.

### Water samples

The presence of rotavirus, enterovirus and astrovirus, as well as the abundance of indicator bacteria in the water source and in water used for irrigation was determined from samples obtained during the cold-dry (November to February) and warm-rainy (May to October) seasons in 2001 and 2002. These seasonal categories were defined according to two meteorological parameters: temperature and rainfall [21,22]. Samples from water used for irrigation were obtained from ten sampling points, randomly selected from a regular grid of 250 observation points covering the Xochimilco canal network, which had been set up for previous studies in the area [23]. For viral detection, a 20 L volume was collected at each sampling point for each season per year. Samples for bacteriological analyses were collected at a depth of 40 cm in 1 L sterile polypropylene flasks.

Water source samples were obtained from ten wells randomly selected from the total of 60 wells that form part of the Mexico City water supply system. Samples were taken directly from the wells prior to chlorine disinfection. For each season and year, 1200 L of water was filtered through a 1 MDS electropositive filtering cartridge at each well (CUNO, Meriden, CO). Within six hours of sampling, the cartridges were transported cold (4°C) to the laboratory. For bacterial analyses, 1 L samples were taken in sterile polypropylene containers. At each sampling point, pH, temperature and conductivity were measured using a portable YSI 3500 pH-conductivity meter (Yellow Spring, OH) and dissolved oxygen measured with an YSI 51B oxygen meter (Yellow Spring, OH). In the laboratory, the 80 water samples (10 samples from irrigation water and 10 water source samples, both taken each season for two years) were analyzed for the following enteric viruses: enterovirus (EV); rotavirus (RV); and astrovirus (AST); and for indicator organisms including total coliform (TC), faecal coliform (FC), and enterococci (FE), as described below.

### RNA extraction and cDNA synthesis

Water samples were filtered through electropositive Virosorb 1 MDS cartridges (CUNO, Meriden, CO). Once water samples were concentrated to a 30 mL volume, RNA was extracted using a Trizol LS reagent (Invitrogen, Carlsbad, CA) and chloroform. Aliquots of 300 μL of water were mixed with 300 μL of PBS 1× and shaken vigorously five times, leaving the vials on ice for one minute between each shaking, and then centrifuged at 12,000 × g for five minutes. The upper phase containing RNA was transferred and 500 μL of Trizol added, gently mixing for one minute before replacing on ice. This procedure was repeated five times. Subsequently, 100 μL of chloroform was added gently and shaken vigorously five times.

Following centrifugation at 12,000 × g for five minutes, the upper phase was recovered and incubated with the same volume of isopropanol at 4°C for 30 minutes, and then centrifuged for 15 minutes at 12,000 × g at 4°C. The pellet was washed with 1 mL of absolute ethanol and centrifuged for a further five minutes at 12,000 × g at 4°C. Finally, the pellet was dried at room temperature and re-suspended in 20 μL RNAse free water, and stored at -70°C until RT-PCR analysis took place.

cDNA synthesis (RT reaction) was performed in a 20 μL reaction volume containing 1 μL of RNA, 1 μL of 5 pM primer, and 9.9 μL nuclease-free water (Invitrogen, Carlsbad, CA) at 70°C for 10 minutes. Subsequently, 8.1 μL of a mix containing 4 μL of 5× first strand buffer [250 mM Tris-HCl (pH 8.3), 375 mM KCl, 15 mM MgCl_2_], 2 μL 0.1 M DTT, 2 μL 5 mM dNTPs and 20 U Super Script II reverse transcriptase (Invitrogen) was added. The reaction was carried out at 42°C for one hour and subsequently at 70°C for 15 minutes.

### cDNA amplification

Polymerase chain reaction (PCR) was carried out in a 25 μL volume with a mix of 17.27 μL nuclease-free water, 2.5 μL 10× buffer [100 mM Tris-HCl (pH 8.3), 500 mM KCl, 15 mM MgCl_2_, 0.01% w/v gelatin], 1.6 μL 5 mM dNTPs, 1 μL of 25 pM of each primer, and 0.625 U of Ampli Taq Polymerase (Roche). The primers used to amplify the conserved region for group A that codes for the VP7 structural protein of rotavirus (RV) were as follows: forward (5-GGCTTTAAAAGAGAGAATTTCCGTCTGG-3) and reverse (5-GATCCTGTTGGCCATCC-3) [24], for enterovirus (EV) the highly conserved region among picornaviruses 5'NCR forward (5-TCCGGCCCCTGAATGCGG-3) and reverse (5-CACCGGATGGCCAATCCAAT-3) [24], and for astrovirus (AST) the conserved region of ORF2 forward (5-GGTGTCACAGGACCAAAACC-3) and reverse (5-TTAGTGAGCCACCAGCCATC-3) [25].

The amplification conditions included denaturation at 94°C for one minute and 33 cycles at 94°C for 30 seconds, 50°C for 30 seconds and 72°C for 25 seconds, with a final elongation at 72°C for seven minutes. Agarose gels were stained with ethidium bromide and examined under ultraviolet light.

### Bacteriological water analyses

Bacteriological analyses of TC, FC, and FE were carried out according to the membrane filtration method using selective media and following standard procedures [26]. The bacteriological culture media used were m-Endo (BBL), m-FC (BBL) and KF-Streptococcus Agar (BBL) according to manufacturer's instructions for TC, FC and FE respectively. Briefly, 1 L water samples were taken in sterilized polypropylene bottles. The samples were transported to the laboratory under cold conditions (4°C) and processed in the within 6 hours of sampling at the most. When necessary samples were diluted, mainly for irrigation water, while 100 mL of drinking water samples were directly filtered. After filtration through a 0.45 μm nitrocellulose membrane (Millipore), the media plates were incubated at 36° for 24 h for TC, at 44.5°C for 24 h for FC and at 36°C for 48 h for FE.

### Detection of Coliphages

Coliphages were detected from concentrated water samples using *Escherichia coli *K12 Hfr (ATCC) as the host bacterium according to the double layer agar method. Briefly, 5 mL of Trypticase peptone semisolid agar (1%) containing 500 μL of K12 in exponential growth phase and 500 μL of concentrated water sample were poured onto Trypticase peptone solid agar. Plates were incubated at 37°C for 18 h and the coliphage plaques counted.

### Statistical analysis

Data were analyzed using a generalized linear model approach [27,28]. First, we tested for differences between years, between seasons and between seasons within each year (year X season interaction) in the occurrence of viruses or the abundance of indicator bacteria. For viruses, the response variable (presence/absence) was assumed to follow a Bernoulli distribution; the abundance of bacteria was assumed to follow a quasiPoisson distribution (to compensate for overdispersion when a Poisson distribution was used). A full factorial model with factors Year (2001 vs. 2002) and Season (cold-dry vs. warm-rainy) was fitted to each response variable (occurrence of rotavirus, enterovirus and astrovirus; abundance of TC, FC, and FE) separately. The terms' significance was judged with basis on the change in deviance that its deletion from the model produced, which approximately follows a Chi-square distribution [27].

Second, in order to examine the relationship between the occurrence of viruses or the abundance of bacteria and the physicochemical environmental variables recorded, log-linear (for bacteria abundance, with a quasiPoisson distribution) or logit (for viruses occurrence, with a Bernoulli distribution) models were separately fitted to each response variable (abundance of TC, FC, and FE; occurrence of rotavirus, enterovirus and astrovirus) with the environmental variables (temperature, conductivity, pH and Oxygen concentration) as predictors; for viruses' models, the abundance of indicator bacteria (i.e., TC, FC and FE) were also included as predictors. In each case, predictor variables significantly related to the response variable were identified also with basis on the change in deviance that its deletion from the model produced.

Finally, simple two-way analyses of variance were used to test for differences between years, between seasons and between seasons within each year in the means of the physicochemical environmental variables recorded (pH, Oxygen concentration, temperature and conductivity).

## Results

### Water used for irrigation

**Figure 1:**
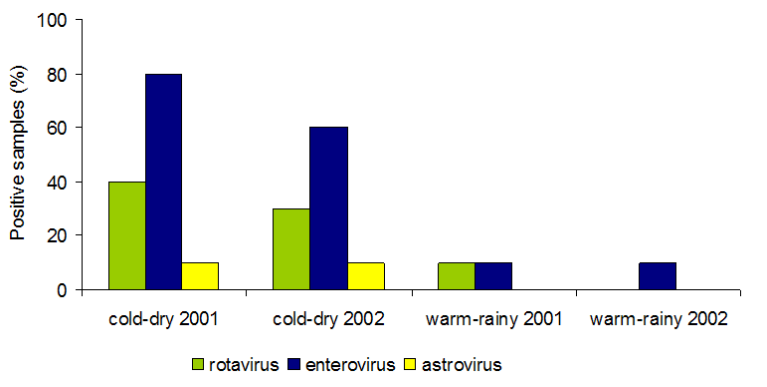
**Percentage of RT-PCR positive samples**. Rotavirus (RV), enterovirus (EV) and astrovirus (AST) in water used for irrigation over annual cycles (2001 and 2002). The Astrovirus genome was not detected either of the two warm-rainy seasons, and the genome rotavirus was not detected during the 2002 warm-rainy.

Deviance analysis resulting from the generalized linear model approach showed no significant differences between years, between seasons, nor between seasons within each year in terms of AST presence. However, there were significant differences between seasons when the presence of both EV and RV were considered. The presence of these pathogens was significantly more frequent during the cold-dry season (0.75 and 0.35, respectively) than in the warm-rainy season (0.10 and 0.05, respectively) (Figure 1).

**Figure 2:**
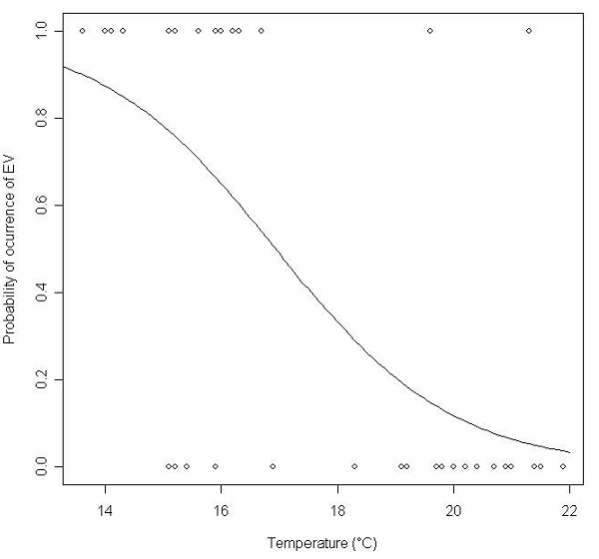
**Relationship between the occurrence of EV in irrigation waters and temperature**. Points represent observed values (1 = presence, 0 = absence), while the continuous curve represents the logit model describing relationship between the probability of occurrence of EV and temperature: P = exp (11.1268 - 0.657*Temperature)/(1 + exp(11.1268 - 0.657*Temperature)).

The presence of AST and RV showed no significant relationship with either the environmental variables recorded (pH, temperature, conductivity and dissolved oxygen concentration) or with the abundance of bacterial indicators. By contrast, EV presence was significantly related to temperature (Figure 2) but not to the abundance of any of the bacterial indicators.

**Figure 3:**
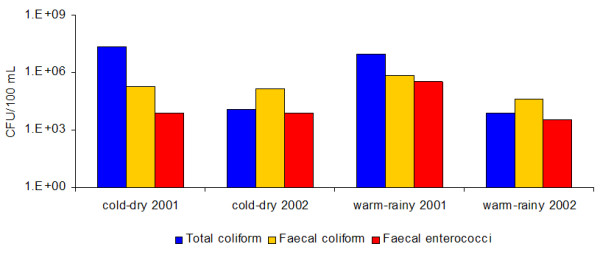
**Indicator bacteria in irrigation water**. Counts of total coliforms (TC), faecal coliforms (FC) and enterococci (FE) in water used for irrigation over two seasons each year. Bacterial counts were determined by filtration membrane method and standard culture media.

As for bacterial indicators, all samples were positive for the three bacterial groups, indicating continuous faecal contamination of the water used for irrigation. Deviance analysis showed no significant differences between years, between seasons, or between seasons within each year when the abundance of faecal coliform (FC) or enterococci (FE) were considered. However, the abundance of these bacterial groups was significantly related to pH. By contrast, there were significant differences in the abundance of TC between years and between seasons. TC was significantly more abundant in 2001 than in 2002, and during the dry season than during the rainy season (Figure 3). However, the abundance of TC was not significantly related to any of the environmental variables recorded (pH, temperature, conductivity and dissolved oxygen concentration).

Table 1 shows the association between enteric virus, coliphages and indicator bacteria when detected in irrigation water. This association is clear as reflected by the *p-value*. Positive samples for coliphages coincided more frequently with the positive samples for indicator bacteria than EV, or the other enteric viruses.

**Table 1:** Association of enteric viruses, bacteria and coliphage in irrigation water.

	Coliphages				
		
	Negative (n = 40)		Positives (n = 40)		
			
Microorganisms	n	%	N	%	*p value*
TC	1	2.5	33	82.5	0.0003
FC	0	0	35	87.5	0.0000
FE	0	0	40	100	0.0000
EV	3	7.5	16	40	0.0182
RV	7	17.5	7	17.5	0.1502
AST	7	17.5	2	5	0.4587

### Water used for drinking

No viruses were detected in the drinking water wells prior to chlorination. According to the physicochemical parameters: pH; temperature; dissolved oxygen concentration; and conductivity; there were no differences in prevailing conditions for each sampling station. This demonstrated that there was no seasonal difference in the conditions pertaining to these sources of drinking water.

Indicator bacteria were detected in the pre-chlorinated drinking water samples. Results of TC, FC, and FE presence (Table 2) show that FE were most frequently isolated and most abundant, with five positive samples in 2001 and 13 positive samples in 2002. In terms of FE abundance, deviance analysis showed no significant difference between years, between seasons or between seasons within each year. Similarly, no significant relationship was found between the abundance of FE and any of the environmental variables (pH, temperature, conductivity and dissolved oxygen concentration). There were significant differences between years and between seasons in both TC and FC abundance. These bacteria types were significantly more abundant in 2002 than in 2001, and showed a higher presence during the dry season than the rainy season. TC and FC abundance were also significantly related to variation in conductivity.

**Table 2:** Total bacterial counts (CFU/100 mL) for TC, FC and FE in the water source.

Year/season	TC		FC		FE	
	**+/n**	**CFU/100 mL**	**+/n**	**CFU/100 mL**	**+/n**	**CFU/100 mL**
	
2001						
warm-rainy	0/10	0	1/10	1	1/10	1
cold-dry	1/10	1	0/10	0	4/10	101
2002						
warm-rainy	2/10	24	1/10	1	7/10	370
cold-dry	4/10	404	4/10	53	6/10	216

+/n positive samples with respect to total number of samples

### Physicochemical parameters

The physicochemical parameters recorded were temperature, conductivity, pH, and dissolved oxygen. Average values for each season, year, and water type are shown in Table 3.

**Table 3:** Physicochemical parameters registered in water samples.

Parameter	2001		2002	
	**Warm-rainy**	**Cold-dry**	**Warm-rainy**	**Cold-dry**
	
**Irrigation**				
Temperature (°C)	20.77	15.26	19.90	15.15
pH	7.53	7.85	7.92	7.78
Conductivity μS/cm)	658.00	711.00	477.20	727.10
Dissolved oxygen (mg/L)	3.27	2.82	3.93	3.94
**Drinking water**				
Temperature (°C)	16.86	15.83	16.02	16.41
pH	6.95	6.78	7.83	7.34
Conductivity (μS/cm)	379.30	315.40	463.30	293.10
Dissolved oxygen (mg/L)	3.48	4.57	3.52	3.09

When water used for irrigation was considered, the two-way variance analysis showed no significant differences between seasons or between years in terms of average pH or average dissolved oxygen concentration. By contrast, average temperature during the cold-dry season was significantly (p < 0.001) lower than during the warm-rainy season.

Significant variations between seasons within each year in terms of average conductivity were also found; in 2001 there were no significant differences between seasons but, in 2002, average conductivity was significantly higher during the cold-dry season suggesting that there was a variation between years for some parameters.

For water source samples, analyses showed no significant variations in terms of average temperature. By contrast, there were significant differences between years (p < 0.001) and between seasons (p < 0.01) in terms of average pH. Significant variations between seasons within each year for average dissolved oxygen concentration were also found. In 2001, average conductivity was significantly higher during the warm-rainy season, whereas in 2002 there were no significant differences between seasons. Finally, average conductivity was significantly higher (p = 0.011) during the cold-dry seasons.

## Discussion

The Mexico City area, where the study was carried out has been classified as a tropical highland [29], with an altitude of 2240 masl and climatic conditions, with a cold-dry autumn and winter followed by a warm-dry spring and a warm-wet summer.

The current study used two basic meteorological parameters to define the seasonal categories (cold-dry and warm-rainy); temperature and rainfall as reported by the official National Meteorological System (Sistema Meteorológico Nacional) [21,22].

Treated wastewater represents the most important input to the aquatic system; a process that does not consider virus elimination. There are also raw wastewater discharges directly from households (2,015 houses with about 4.5 persons) that a lack sewer system [30] and it is estimated that 2,116 kg of faeces enter the aquatic system daily. Due to the continuous faeces contribution and enteric virus detection predominantly in the cold-dry season, a seasonal pattern regarding viral presence is suggested. On the other hand, studies of diarrheal disease caused by rotavirus and astrovirus in young children from Southern Mexico City [14,31] showed higher rates in the autumn and winter months [14]. This increased incidence reflects the findings of the current study in which viral presence is higher in the cold-dry season.

Unfortunately in Mexico, the National Epidemiological Surveillance System [32] does not report epidemiological information that would indicate the actual number of viral gastroenteritis cases, nor their seasonal behaviour. This is despite the fact that gastrointestinal disease at a local level (Mexico City) is among the 20 main causes of hospitalization [33].

During the cold-dry season the average low temperature was 4°C with an average precipitation of less than 10 mm [21,22], which had an effect on the presence of both bacteria and enteric viruses. This is related to lower water levels, higher concentration of organic matter [23], and lower temperature [34], the latter favouring the presence of enteric viruses [35]. These measurements of temperature and precipitation support the current study for EV and RV, which showed a higher presence during the cold-dry season. Conversely, AST was present in only 10% of the samples. The variation between the frequencies of different viruses can be associated with structural virus characteristics, in that the RV capsid presents three protein layers, as compared with only one for AST. The higher frequency of EV could be related to the massive polio vaccination campaigns that are carried out in Mexico three times a year for children of less than five years old. In previous studies, polio vaccine was isolated from both wastewater and river water two or three following the vaccination campaign [36-38]. Other studies are needed to ensure that the EV detected in the water samples used for irrigation corresponds to the vaccine type.

In the warm-rainy season, the temperature can reach an average of 24°C [21,22], while rainfall can reach 1,500 mm. At the beginning of the rainy season, two natural processes are evident in the canal system: soil washing and water dilution. These promote an increase in bacterial density and counts in water, while towards the middle of the rainy season, bacterial density decreases due to dilution. Although warm-rainy temperature favours bacterial growth, enteric viruses could be damaged by rising temperatures, as proved previously, when EV and RV were studied in fresh water at 22°C and 20°C [39,40]. EV, RV and AST were practically absent during the warm-rainy season in both years.

The rainfall, plus a significant increase in temperature compared with that of the cold-dry season, contributes to the presence of these viruses in the water used for irrigation from this tropical high-altitude area. Additionally, solar radiation, especially UVB (320-280 nm), has recently been reported as an important parameter that affects viral presence and infectivity [41,42], another environmental parameter that should be included in future studies.

It is important to point out that TC is a group that includes enteric and non-enteric bacteria [43], and the lower TC counts could be related to interference of non-coliform bacteria that inhibit coliform bacteria growth, as has been shown by Burlingame *et al*. [44], when m-Endo medium was used. Moreover, FC cultivated in m-FC medium at 44.5°C has been reported to promote non-E. *coli *thermophilic growth [45], which can produce a FC overestimation or a false positive reading. The culture media used are those recommended by Standard Methods [26] and also correspond to the official Mexican methods [46] for the enumeration of TC and FC in water samples. However, the use of other methods to measure indicator bacteria that show more specific results, mainly for water from tropical and subtropical areas [45], is recommended for subsequent studies.

The results obtained in this study showed that coliphages can be used as indicators of faecal contamination in reused water, in a complementary role to indicator bacteria. There are publications that support coliphages usefulness as faecal indicators [47-50], because based on their presence it is possible to infer faecal contamination. Our results are in agreement, shown by the significant correlation between coliphages and faecal coliforms.

According with the results, coliphages are useful as index or model organism of the presence of EV, due to the significant relationship showed. These agree with results reported for coliphages and enterovirus [51,52] where there is also highlighted the similarities in physical particle characteristics, as well as resistance to wastewater treatment; which support the idea of using coliphages as enteric viruses index, and also as a process indicator [48].

Coliphages have been shown to be complementary or equivalent to other indicators, therefore it is highly advisable to include them as faecal pollution indicators and also as index of enteric virus for water quality monitoring programs.

Mexico is considered to be a leading country in terms of wastewater recycling [20]. This practice does not appear to be on the wane and it is envisaged that more land will make use of wastewater for irrigation in the future. Mexican regulations [53-55] and World Health Organization guidelines for irrigation [56] consider ≤ 1000 CFU/100 mL coliform bacteria as an acceptable limit for the irrigation of land that is used to grow crops. However, according to the results discussed here, this limit has been exceeded in the study area. The enteric virus and bacterial survival on vegetable surfaces [57], constitutes a serious health risk for agricultural workers, as well as for consumers [58].

Enteric viruses were not detected in the water sources during the seasons and years studied. Nevertheless, the relevance of this area as a source of drinking water makes it important to monitor viral presence regularly. Coliphages may provide adequate viral indicators representing the large group of EV, but further evaluation is required before they can be used for this purpose; tests that were not performed as part of this study due to time and financial constraints.

Indicator bacteria detected in water sources did not show any seasonal trends. The higher FE frequency suggests that these could be better bacterial contamination indicators as compared with TC and FC levels in the region. The water extraction wells are located in the transition area, where sedimentary soil composition is known to favour water infiltration. This can affect groundwater quality because the sewage system is insufficient for the growing population of these areas, and does not exist at all in squatters settlements. Sewer breakages, which are frequent occurrences, could explain the presence of indicator bacteria in the drinking water sources, as well as being important for viral contamination. Groundwater as a source for drinking water presents more stable conditions as compared with surface water; non-solar irradiation and relatively low temperatures are favourable for enteric viral presence and infectivity [18]. In these circumstances, preventive actions should be taken.

## Conclusion

Enterovirus, rotavirus, astrovirus, total coliform, faecal coliform, enterococci and coliphages considered in this study were present in water to be used for irrigation.

The abundant presence of indicator bacteria and enteric viruses in irrigation water proves a continuous raw residual water supply to the aquatic system.

Viral presence in irrigation water, for the specific tropical highland system under study, is similar to that previously reported for temperate zones, during the colder months.

The detection of indicator bacteria in the sources of drinking water shows the contribution of faecal matter in the aquifer and reinforces the need for an adequate disinfection process in order to ensure good water quality in the public supply system.

Analysis to identify the presence of coliphages as indicators of faecal contamination is recommended. These should be considered as complementary to bacterial indicators, and to reflect the general survival conditions of enteric viruses. The fact that coliphages are tolerant to wastewater treatment makes them suitable indicators for the evaluation of recycled water to be used for irrigation and recreational purposes.

This low-cost strategy of using viral and bacterial indicators to confirm water quality for drinking and irrigation is attractive and advisable for low income countries, reflected in a public health benefit.

## Abbreviations

AST: astrovirus; cDNA: complementary DNA; EV: enterovirus; FC: faecal coliform; FE: enterococci; NCR: non code region; ORF: open reading frame; PCR: polymerase chain reaction; RT: reverse transcriptase; RT-PCR: reverse transcriptase-polymerase chain reaction; RV: rotavirus; TC: total coliform; UVB: ultraviolet B radiation.

## Competing interests

The authors declare that they have no competing interests.

## Authors' contributions

ACE and MMH conceived and designed this study, performing some preliminary assays. ACE contributed to the acquisition of field and experimental data, and carried out the analytical work. ACE initiated data interpretation, and drafted and revised the manuscript. MMH and CFA offered analytical suggestions, assisted with the interpretation of results, and made critical revisions to the manuscript suggesting details for the final draft. SSC contributed to statistical analyses and interpretation of data. All authors read and approved the final manuscript before submission.
